# Coverage and equity of essential care services among stroke survivors in the Western Province of Sri Lanka: a community-based cross-sectional study

**DOI:** 10.1186/s12913-022-08404-5

**Published:** 2022-08-10

**Authors:** Nalinda Tharanga Wellappuli, Hettiarachchige Subashini Rasanja Perera, Thashi Chang, Gunendrika Kasthuriratne, Nalika Sepali Gunawardena

**Affiliations:** 1 Centre for Health Economics & Policy Innovation (CHEPI), Imperial College Business School, South Kensington Campus, London, SW7 2AZ UK; 2 Ministry of Health, ‘Suwasiripaya’, No-385, Rev. Baddegama Wimalawansa Thero Mawatha, Colombo-10, Sri Lanka; 3 Department of Clinical Medicine, Faculty of Medicine, University of Colombo, No- 25, Kynsey Road, Colombo 08, Sri Lanka; 4 Department of Rheumatology and Rehabilitation, National Hospital, E.W. Perera Road, Colombo-10, Sri Lanka; 5 World Health Organization-Country Office for Sri Lanka, No- 5 Anderson Road, Colombo 05, Sri Lanka

**Keywords:** Stroke, Follow up care, Coverage of Services, Sri Lanka, Non-Communicable Diseases (NCD), Public Health Policy, Universal Health Coverage

## Abstract

**Background:**

Stroke survivors require continuing services to limit disability. This study assessed the coverage and equity of essential care services received during the first six months of post-stroke follow-up of stroke survivors in the Western Province of Sri Lanka.

**Methods:**

A multidisciplinary team defined the essential post-stoke follow-up care services and agreed on a system to categorize the coverage of services as adequate or inadequate among those who were identified as needing the said service. We recruited 502 survivors of first ever stroke of any type, from 11 specialist hospitals upon discharge. Six months following discharge, trained interviewers visited their homes and assessed the coverage of essential services using a structured questionnaire.

**Results:**

Forty-nine essential post-stroke follow-up care services were identified and categorized into six domains: monitoring of risk conditions, treatment, services to limit disabilities, services to prevent complications, lifestyle modification and supportive services. Of the recruited 502 stroke survivors, 363 (72.3%) were traced at the end of 6 months. Coverage of antiplatelet therapy was the highest (97.2% (*n* = 289, 95% CI 95.3- 99.1)) while referral to mental health services (3.3%, *n* = 12, 95% CI 1.4–5.1) and training on employment for the previously employed (2.2%, *n* = 4, 95% CI- 0.08–4.32), were the lowest among the six domains of care. In the sample, 59.8% (95% CI 54.76–64.48) had received an ‘adequate’ level of essential care services related to treatment while none received an ‘adequate’ level of services in the category of support services. Disaggregated service coverage by presence and type of limb paralysis within the domain of services to prevent complications, and by sex and education level within the domain of education level, show statistically significant differences (*p* < 0.05).

**Conclusions:**

Apart from treatment services to limit disabilities, coverage of essential care services during the post-stroke period was inadequate. There were no apparent inequities in the coverage of vast majority of services. However focused policy decisions are required to address these gaps in services.

## Background

Stroke is characterised by rapidly developing focal or global disturbance of cerebral function lasting 24 h or more or leading to death, with no apparent cause other than the vascular origin [1]. It has been estimated that annually, 15 million suffer from strokes globally, and of those, 5 million die and 5 million become permanently disabled [2]. Stroke is the second highest ranked disease to contribute toward disability- adjusted life years [3]. Following acute care of stroke, it has been found that most of the survivors need follow-up care [4, 5]. Post-stroke follow-up care addresses issues of physical health, mood, cognition, and activities of daily living [6]. It also includes secondary prevention of stroke by controlling modifiable risk factors and tertiary prevention by detection and management of stroke- related impairments. However, gaps in post-stroke follow-up care have been identified even in developed countries [7].

Sri Lanka, an island situated just south of the Indian subcontinent with a population of approximately 22 million, is estimated to have one of the highest age-standardised stroke mortality and stroke mortality-to-incidence ratios [8] and reports a crude stroke prevalence of 10.4 per 1000 adults [9]. Even though Sri Lanka is a country with a pluralistic health care system, the vast majority of Sri Lankans seek care from public sector allopathic healthcare institutions where care is delivered free of charge [10]. In the allopathic system, acute care for stroke is provided mainly through 122, secondary or tertiary level hospitals [11]. The majority of stroke survivors are then followed up at the same institution or in another institution closer to the patient’s residence. Essential post-stroke follows up care services for stroke survivors in Sri Lanka are known to be non-uniform and the majority of institutions function with limited facilities for rehabilitation of stroke survivors. Even though currently community-based nursing officers and public health fields staff employed in provision of maternal and child health services and some selected NCD screening and preventive services, they are not entrusted in provision of essential post stroke follow up care services in Sri Lanka.

Sri Lanka is reporting increasing numbers of stoke cases and a systematic assessment of care service provision has not been conducted. As health policy authorities of Sri Lanka planned a review to design models of service delivery to achieve Universal Health Coverage, it was timely to have data and information for necessary policy options. To date, the coverage of essential post-stroke follow-up care services of stroke survivors has not been studied in Sri Lanka. Therefore, we aimed to determine the proportion of stroke survivors who received essential post-stroke follow up care services in the Western Province of Sri Lanka and to explore any inequities in coverage of essential post-stroke follow-up health services among stroke survivors. Conducting this study in Western Province, which consists of better resourced Colombo, Kalutara and Gampaha districts compared to rest of the country, will be an eye opener on the status of post-stroke follow up-care services in Sri Lanka.

## Methods

### Study design and study population

We conducted a descriptive cross-sectional study to assess the coverage of essential post-stroke follow-up care services. We defined the study participants as stroke survivors with documented evidence of first-ever stroke of any type who have reached 6 months following the stroke episode. The estimated sample size based on an assumption that only 50% of stroke survivors received a good level of defined essential post-stroke follow up care services, with an added non-response rate of 10% and a loss to follow-up rate of 20% was 500. Provision for loss to follow-up was because the provisional recruitment of study participants was done at the hospitals at the time of discharge after the acute stage while they were met at their residences at the end of six months of data collection. High mortality due to stroke in the immediate post-stroke period was the other reason for the provision of loses to follow-up.

Eleven secondary and tertiary level hospitals in the three districts of the Western province were considered as the sites to recruit potential study participants and the numbers recruited from each of the hospitals were based on the probability proportionate to the number of live discharges of stroke patients from each hospital.

### Defining essential post stroke follow up care services

We consulted a group of experts in the fields of Neurology, Rheumatology, Psychiatry, Clinical Medicine, Community Medicine, Nursing, Physiotherapy, Occupational Therapy, and Speech and Language Therapy and they identified and defined the essential post-stroke follow-up care services to be delivered within the first six months of the first-ever stroke incident in per the resource availability within Sri Lanka. They also grouped the defined essential post-stroke follow-up care services into six domains of care as follows: monitoring of risk conditions, treatment, services to limit disabilities, services to prevent complications, lifestyle modifications and supportive services. These domains consisted of 10,5,16,7,7, and 4 essential post-stroke follow-up care services within each respectively as listed in Table 3.

### Study instrument

An interviewer-administered study instrument was developed to obtain the information related to the receipt of these services within the post-stroke period irrespective of the type/sector of the healthcare provider and related information to assess the coverage and access to services were equitable. Part 1 of the questionnaire consisted of questions related to the recruitment of stroke survivors at the hospital. This includes questions on basic socio-demographic information, telephone numbers, postal address, and a section to draw a sketch to the stroke survivors’ usual residence. Part 2 of the interviewer-administered study instrument was completed, after six months of the acute stroke incidence by visiting the residence of the stroke survivor. It consisted of questions related to receipt of essential post-stroke follow-up care services identified. We consulted a multidisciplinary expert group in identifying the most suitable mode/source of information for clarifying the receipt of services. The group recommended 3 data sources: ask from the patient, ask from caregivers, and verify from medical records. The study instrument specified the source/s for each question. The multidisciplinary group identified some services that may not regularly be recorded in the medical records and advised to crosscheck those with the patient and caregiver if no written records were found.

### Data collection

The study was conducted during the period of May 2017 to February 2018 and the data was collected at two points in time. Firstly, by recruiting stroke survivors at the hospital and thereafter by visiting the home after of six months from the time of the stroke incident. Eleven post-intern medical officers were selected for the data collection for the recruitment of stroke survivors. The research team conducted a 3-h training session for selected data collectors on identifying suitable study participants, obtaining informed consent, and administering the questionnaire. Data collectors visited all the wards that are likely to treat stroke patients’ and identified the stroke patients that had been admitted. They maintained a list and were tracking their progress and identified the planned date of discharge. The data collectors then approached them 1–2 days before discharge and invited them to participate in the study by verifying the inclusion criteria. Data collectors completed the data collection by interviewing the identified stroke survivors or their bystanders at the hospital before discharge. Collection of data by visiting the home of the study participants six months from the time of the stroke incidence was done by a team of trained pre-intern medical graduates. Each of the trained data collectors was allocated a specified list of study participants in a specified geographical location to be visited. The list indicated the dates by which the study participants should be visited, and the data collectors called the telephone numbers given by the study participants to locate and request an appointment to visit the study participants. The preferred telephone number was used for this purpose. The appointments were taken to ensure the availability of the stroke survivor and a regular caregiver. If the patients were not contactable through the specified telephone numbers, the data collectors were advised to visit the area by using the address and sketched map to locate the study participants. The training program for these medical graduates included theoretical background related to the services identified, as they had to explore the validity of answers given by the data collectors.

### Data analysis

Based on the clinical presentation, the essential post-stroke follow-up care services that each individual study participant should receive under each domain of care were identified out of the defined 49 services. For example, patients without diabetes mellitus, were not considered eligible to receive anti-diabetic medication. Accordingly, the proportion of essential post-stroke follow-up care services received by each study participant was then estimated.

Study participants who had received more than or equal to 75% of the services they should have received were categorised as having received an ‘adequate’ proportion of health services, while the rest were categorised as ‘inadequate.

The proportion of study participants who received adequate and inadequate levels of indicated essential post-stroke follow-up care services within each domain of care, was further disaggregated using selected equity dimensions to explore any inequalities in coverage of essential post-stroke follow-up care services [12]. The selected equity dimensions were sex, age, education level, marital status, family income and presence and the type of paralysis of the limb.

The ethical clearance was obtained from the Ethics Review Committee of the National Institute of Health Sciences- Sri Lanka. Data collectors obtained informed written consent from the identified stroke survivors by providing all relevant information and obtaining informed written consent. In the context of the patient not being able to provide consent due to a medical reason proxy consent was obtained.

## Results

Out of the 502 study participants selected, 139 did not participate in the study giving a response rate of 72.3%. Of the 139 non-respondents, 97 (19.3%) had died and 39 (7.8%) study participants could not be contacted and 03 (0.6%) withdrew consent. A majority of the stroke survivors (*n* = 301, 82.9%) were able to respond to the interviewer-administered study instrument by themselves without the assistance of a caregiver/bystander. The mean age of the study sample was 64.8 years (SD = 11.5 years, range 34 – 92 years).

The socio-demographic characteristics of the study population are shown in Table 1.

**Table 1:** Distribution of the Study participants characteristics

Characteristic		(*N* = 363)	%
Age category (In years)	21–40	9	2.4
	41–60	116	32.0
	61–80	210	57.9
	81–100	28	7.7
Sex	Male	202	55.6
	Female	161	44.4
Marital status	Married	276	76.0
	Unmarried	11	3.0
	Divorced	2	0.6
	Widowed	71	19.6
	Separated	3	0.8
Highest level of education obtained	No schooling	18	5.0
	Year 1–5	85	23.4
	Year 6–8	92	25.3
	Year 9–11	68	18.7
	GCE O/L passed*	68	18.7
	GCE A/L passed**	30	8.3
	Other	2	0.6
Family monthly income (Sri Lankan Rupees) *N* = 289	≤ 25,000	109	37.7
	25,001–50,000	132	45.6
	50,001–75,000	30	10.4
	> 75,000	18	6.3

*General Certificate of Examination - Ordinary Level

**General Certificate of Examination - Advanced Level

Most strokes were ischaemic (*n* = 289, 79.6%) with a majority presenting with hemiplegia (*n* = 292, 80.0%).

Table 2 describes the Risk factors and Comorbidities among the study participants.

**Table 2:** Distribution of common risk factors and comorbidities in the sample

Risk factors and Comorbidity	*N* = 363	Percentage
Hypertension	222	61.2
History of alcohol consumption	143	39.4
Diabetes Mellitus	128	35.3
History of smoking	120	33.1
Hyperlipidemia	116	32.0
Family History of Stroke	71	19.6
Ishaemic Heart Disease	56	15.4
Obesity	44	12.1
Congestive Cardiac Failure	40	11.4
History of Transient Ishaemic attack	31	8.5
History of Valvular heart disease	17	4.7
Atrial Fibrillation	11	3.0

The commonest risk factor/co-morbidity among the study sample was hypertension (*n* = 222, 61.2%) and previously diagnosed atrial fibrillation was the least common ( *n* = 11, 3.0%) risk factor (Table 2).

Table 3 describes the percentage of stroke survivors that received the required essential post-stroke follow-up care services according to the identified six categories. Anti-platelet therapy was the most received service with 97.2% (*n* = 289, 95% CI 95.3- 99.1) of ischaemic stroke survivors receiving it. The monitoring of blood pressure at least once was performed on 93.9% (*n* = 341, 95% CI 91.4- 93.9) but the regular monitoring of blood pressure once a month among hypertensives (*n* = 222) was only among 27% (*n* = 60, 95% CI 21.1–32.8) of stroke survivors. More than half of the study sample were screened for diabetes and dyslipidaemia but mostly not followed up. Services to limit disabilities were not received by more than 50% of stroke survivors, apart from early mobilisation and walking training which were received by 59.3% (*n* = 176, 95% CI 53.7–64.8).

**Table 3:** Proportions of stroke survivors who received follow-up care

Service	n	% (out of those who should have received the service)	95% CI
**Monitoring of Risk Conditions**			
Blood pressure measured at least once (*N* = 363)	341	93.9	91.4- 93.9
Blood pressure measured 6 times or more among study sample with elevated blood pressure (*N* = 222)	60	27.0	21.1–32.8
Blood lipids monitoring at least once (*N* = 363)	184	50.7	45.5–55.8
Blood lipids monitoring 2 times or more among study sample with elevated blood lipids (*N* = 116)	14	12.1	6.1–18.0
Blood glucose monitoring at least once (*N* = 363)	226	62.3	57.3–67.2
Blood glucose monitoring 6 times or more among the study sample with elevated blood sugar levels (*N* = 128)	9	7.0	2.6–11.4
Body Mass Index monitoring (*N* = 363)	57	15.6	11.8–19.3
2-D Echocardiogram performed (*N* = 363)	187	51.5	46.3–56.6
Electrocardiograph (ECG) performed (*N* = 363)	316	87.1	83.6–90.5
Carotid duplex performed among those with ischemic stroke (*N* = 289)	79	27.3	22.1–32.4
**Treatment services**			
Anti-hypertensive treatment for study sample with elevated blood pressure (*N* = 222)	164	73.9	68.1- 79.7
Lipid lowering drugs for the study sample with elevated blood lipids (*N* = 116)	61	52.6	43.5- 61.7
Blood glucose lowering treatment for study sample with elevated blood glucose levels (*N* = 128)	82	64.1	55.7–72.3
Drugs for management among those with atrial fibrillation (*N* = 26)	10	38.5	19.8–57.2
Anti-platelet treatment for the study sample with ischemic stroke (*N* = 289)	281	97.2	95.3–99.1
**Services to Limit Disabilities**			
Early mobilization before discharge from hospital for study sample with lower limb paralysis but not quadriplegia (*N* = 297)	176	59.3	53.7–64.8
Advice on walking training and relevant limb exercises at least once for study sample with lower limb paralysis but not quadriplegia (*N* = 297)	176	59.3	53.7–64.8
Training on moving from bed to chair and vice versa for study sample with lower limb paralysis but not quadriplegia (*N* = 297)	139	46.8	41.1–52.4
Screening for vision impairment at least once (*N* = 363)	97	26.7	22.1–31.2
Screening for cognitive impairment at least once (*N* = 363)	25	6.9	4.2–9.5
Referral speech and language therapy clinics for study sample with speech difficulties (*N* = 203)	51	25.1	19.1–31.0
Training on activities of daily living at least once (*N* = 363)	49	13.5	9.9–17.0
Provision of modified equipment’s to support activities of daily living (*N* = 363)	12	3.3	1.4–5.1
Advice to modify houses to limit disabilities at least once (*N* = 363)	65	17.9	13.9–21.8
Referral to training on swallowing for those with swallowing difficulties group (*N* = 98)	29	29.6	20.5–38.6
Swallowing training once a month for those with swallowing difficulties group (*N* = 98)	19	19.4	11.5–27.2
Advice on managing NG tube at least once for the NG tube users (*N* = 93)	32	34.4	24.7–44.0
Advices on managing urinary catheter at least once for urinary catheter users (*N* = 92)	39	42.4	32.3–52.5
Referral to closet hospital for catheter management for urinary catheter users (*N* = 92)	27	29.3	20.0–38.6
Training on pelvic floor exercises at least once for those with urinary incontinence (*N* = 97)	13	13.4	6.6–20.1
Advice on sexual activities at least once among those who considered it as relevant (*N* = 98)	7	7.1	2.0–12.1
**Services to Prevent complications**			
Advised on use of compression stockings for the study sample with lower limb paralysis (*N* = 303)	23	7.6	4.6–10.5
Advice on family planning at least once for 15–49 years females, who were declared it relevant (*N* = 10)	2	20	0–44.7
Advice to prevent shoulder dislocation at least once for the study sample with shoulder pain (*N* = 149)	77	51.7	43.6–59.7
Referral for mental health screening at least once (*N* = 363)	12	3.3	1.4–5.1
Advising at least once on positioning to prevent pressure ulcers for study sample with lower limb paralysis (*N* = 303)	123	40.6	35.0–46.1
Advising at least once on falls prevention (*N* = 363)	205	56.5	51.4–61.6
Advising on the use of walking aids to prevent falls (*N* = 363)	101	27.8	23.1–32.4
**Services to Modify Lifestyle**			
Inquiring about smoking habits at least once (*N* = 363)	211	58.1	53.0–63.2
Advising on prevention of smoking at least once for those who were inquired on smoking habits (*N* = 211)	178	84.4	79.5–89.3
Inquiring about alcohol consumption habits at least once (*N* = 363)	208	57.3	50.6–64.0
Advising on prevention of alcohol use at least once for those who were inquired on alcohol consumption habits (*N* = 208)	177	85.1	79.9–90.3
Advising on healthy diet at least once (*N* = 363)	170	46.8	39.3–54.3
Providing written instructions on healthy diet at least once for those who were provided with advises on healthy diet (*N* = 170)	28	16.5	2.8–30.2
Referral to nutrition clinic at least once (*N* = 363)	20	5.5	0–15.5
**Services related to Supportive Services**			
Providing information on services by other institutions at least once (*N* = 363)	13	3.6	1.7–5.5
Receiving any benefits from other institutions (*N* = 363)	13	3.6	1.7–5.5
Training on employment, for the previously employed study sample (*N* = 184)	4	2.2	0.1–4.3
Training for the care givers (*N* = 363)	15	4.1	2.1–6.1

Only a minority of stroke survivors (*n* = 12, 3.3%, 95% CI 1.5–5.1) were referred to assess their mental health status. Among those with lower limb paralysis, only 40.6% (95% CI 35.1–46.1) (*n* = 123) had received advice related to positioning to prevent pressure ulcers.

While most of the study sample had been advised to stop smoking (58.1% and 95% CI 53.0–63.2) and consuming alcohol (57.3% and 95% CI 50.6–64.0), only half of the study sample (*n* = 170, 46.8%) had been advised on a healthy diet.

Only 13 participants (3.6%, 95% CI 1.7–5.5) had received information and some benefits such as monthly living allowance, financial aid for improving the home physical environment, self-employment aid, aid equipment from institutions such as the Department of Social Services, Elderly Care Secretariat, and local non-governmental organizations, during the follow-up period. The majority of the caregivers of the participants (*n* = 348, 95.9%) had not received any training related to caregiving during the follow-up period.

Table 4 describes the level of coverage of essential post-stroke follow-up care services received by the individual study participants by identified domains of care. This was calculated by analysing the number of services that study participants received in comparison to the number of services that study participants should receive.

**Table 4:** Distribution of adequate level of essential post stroke follow up care by domains

Level of care in the services received (*N* = 363)	n	%	CI
**Monitoring of risk conditions**			
Adequate	35	9.6	6.6–12.6
Inadequate	328	90.4	86.7–93.1
**Treatment services**			
Adequate	217	59.8	54.7–64.5
Inadequate	146	40.2	35.2–45.8
**Services to Limit Disabilities**			
Adequate	6	1.7	0.4–3.0
Inadequate	357	98.3	96.2–99.3
**Services to Prevent Complications**			
Adequate	18	5.0	2.7–7.2
Inadequate	345	95	92.1–96.9
**Services to Modify Lifestyle**			
Adequate	14	3.9	1.9–5.9
Inadequate	349	96.1	93.5–97.7
**Services related to Supportive Services**			
Adequate	0	0.0	-
Inadequate	363	100	-

The majority (59.8%, 95% CI 54.7- 64.5) of the study participants had received an adequate level of essential post-stroke follow-up care under the treatment services category. However, among other categories, approximately one-tenth of the study participants (9.6%, 95% CI 6.6- 12.6) received an adequate level of essential post-stroke follow-up care services related to the monitoring of risk conditions and 5% of the study participants (95% CI 2.7–7.2) had received an adequate level of care related to services to prevent complications. The adequate care concerning the domains of care to limit disabilities and services to modify lifestyle received 1.7% (95% CI 0.4–3.0) and 3.9% (95% CI 1.9 – 5.9) respectively while none reported receiving adequate supportive care services.

Furthermore, the level of essential post-stroke follow-up care services received, was disaggregated by age, sex, education level, marital status, family income level and presence and type of paralysis of limbs (Table 5) for the domains of care related to the monitoring of risk conditions, treatment services, services to limit disabilities, services to prevent complications, services to modify lifestyle to explore for any inequities in the coverage of services.

**Table 5:** Distribution of equity of level of care by domains of care among stroke survivors

Domains of Care	Adequate level		Inadequate level		Significance	
	N	%	N	%		
**1. Monitoring of risk conditions**						
**Sex category**						
Male	18	8.9	184	91.1	χ2 =	0.2793
Female	17	10.6	144	89.4	df =	1
					*p* =	0.597
**Age category**						
Below 60 years	15	12.0	110	88.0	χ2 =	1.2169
60 years or more	20	8.4	218	91.6	df =	1
					*p* =	0.2699
**Education level**						
0-Grade5	5	4.9	98	95.1	χ2 =	6.0392
Grade 6–11	15	9.4	145	90.6	df =	3
GCE O/L Passed*	10	14.7	58	85.3	*p* =	0.1097
GCE A/L Passed**	5	15.6	27	84.4		
**Marital status**						
Married	31	11.2	245	88.8	χ2 =	3.34
Other	4	4.6	83	95.4	df =	1
					*p* =	0.0675
Family monthly income (LKR)***						
≤ 25,000	11	10.1	98	89.9	Fishers exact test	2.870
25,001–50,000	18	13.6	114	86.4		
50,001–75,000	3	10.0	27	90.0	*p* =	0.395
> 75,000	0	0	18	100.0		
**Presence and type of paralysis of limbs**						
No paralysis	1	2.1	46	97.9	Fishers exact test	3.804
Single limb	2	11.2	16	88.8		
Hemiplegia/Quadriplegia	32	10.7	266	89.3	*p* =	0.092
**2. Treatment services**						
**Sex category**						
Male	124	61.4	78	38.6	χ2 =	0.4844
Female	93	57.8	68	42.2	df =	1
					*p* =	0.519
**Age category**						
Below 60 years	72	57.6	53	42.4	χ2 =	0.3767
60 years or more	145	60.9	93	39.1	df =	1
					*p* =	0.5393
**Education level**						
0-Grade5	59	57.3	44	42.7	χ2 =	3.1121
Grade 6–11	103	64.4	57	35.6	df =	3
GCE O/L Passed*	39	57.4	29	42.6	*p* =	0.3746
GCE A/L Passed**	16	50.0	16	50.0		
**Marital status**						
Married	171	62.0	105	38.0	χ2 =	2.2697
Other	46	52.9	41	47.1	df =	1
					*p* =	0.1319
Family monthly income (LKR)***						
≤ 25,000	70	64.2	39	35.8	χ2 =	3.7407
25,001–50,000	73	55.3	59	44.7	df =	3
50,001–75,000	16	53.3	14	46.7	*p* =	0.2908
> 75,000	8	44.4	10	55.6		
**Presence and type of paralysis of limbs**						
No paralysis	23	48.9	24	51.1	χ2 =	4.7717
Single limb	14	77.8	4	22.2	df =	2
Hemiplegia/Quadriplegia	180	60.4	118	39.6	*p* =	0.0920
**3. Services to limit disabilities**						
**Sex category**						
Male	4	2.0	198	98.0	χ2 = Yates corrected	0.018
Female	2	1.2	159	98.8	df =	1
					*p* =	0.8938
**Age category**						
Below 60 years	4	3.2	121	96.8	χ2 =	2.8072
60 years or more	2	0.8	236	99.2	df =	1
					*p* =	0.0938
**Education level**						
0-Grade5	1	1.0	102	99.0	Fishers exact test	0.957
Grade 6–11	4	2.5	156	97.5		
GCE O/L Passed*	1	1.5	67	98.5	*p* =	0.922
GCE A/L Passed**	0	0.0	32	100.0		
**Marital status**						
Married	5	1.8	271	98.2	χ2 = Yates corrected	0.1784
Other	1	1.1	86	98.9	df =	1
					*p* =	0.6727
Family monthly income (LKR)***						
≤ 25,000	3	2.8	106	97.2	Fishers exact test =	0.532
25,001–50,000	3	2.3	129	97.7		
50,001–75,000	0	0	30	100.0	*p* =	1.000
> 75,000	0	0	18	100.0		
**Presence and type of paralysis of limbs**						
No paralysis	0	0.0	47	100.0	Fishers exact test =	0.406
Single limb	0	0.0	18	100.0		
Hemiplegia/Quadriplegia	6	2.0	292	98.0	*p* =	1.000
**4. Services to prevent complications**						
**Sex category**						
Male	7	3.5	195	96.5	χ2 =	2.1551
Female	11	6.8	150	93.2	df =	1
					*p* =	0.1420
**Age category**						
Below 60 years	10	8.0	115	92.0	χ2 =	3.7418
60 years or more	8	3.4	230	96.6	df =	1
					*p* =	0.0531
**Education level**						
0-Grade5	6	5.8	97	94.2	Fishers exact test =	1.604
Grade 6–11	9	5.6	151	94.4		
GCE O/L Passed*	3	4.4	65	95.6	*p* =	0.716
GCE A/L Passed**	0	0	32	100.0		
**Marital status**						
Married	6	6.9	81	93.1	χ2 = Yates correccted	0.451
Other	12	4.3	264	95.7	df =	1
					*p* =	0.5018
Family monthly income (LKR)***						
≤ 25,000	6	5.5	103	94.5	Fishers exact test =	6.306
25,001–50,000	4	3.0	128	97.0		
50,001–75,000	4	13.3	26	86.7	*p* =	0.68
> 75,000	2	11.1	16	88.9		
**Presence and type of paralysis of limbs**						
No paralysis	9	19.1	38	80.9	Fishers exact test =	15.676
Single limb	0	0	18	100.0		
Hemiplegia/Quadriplegia	9	3.0	289	97.0	*p* =	0.00006
**5. Services to modify life style**						
**Sex category**						
Male	12	5.9	190	94.1	χ2 =	5.3336
Female	2	1.2	159	98.8	df =	1
					*p* =	0.0209
**Age category**						
Below 60 years	7	5.6	118	94.4	χ2 = Yates corrected	0.928
60 years or more	7	2.9	231	97.1	df =	1
					*p* =	0.3355
**Education level**						
0-Grade5	0	0	103	100.0	Fishers exact test =	8.134
Grade 6–11	8	5.0	152	95.0		
GCE O/L Passed*	5	7.4	63	92.6	*p* =	0.028
GCE A/L Passed**	1	3.1	31	96.9		
**Marital status**						
Married	12	4.3	264	95.7	χ2 = Yates corrected	1.111
Other	2	2.3	85	97.7	df =	1
					*p* =	0.2918
Family monthly income (LKR)***						
≤ 25,000	6	5.5	103	94.5	Fishers exact test =	0.943
25,001–50,000	6	4.5	126	95.5		
50,001–75,000	2	6.7	28	93.3	*p* =	0.805
> 75,000	0	0	18	100.0		
**Presence and type of paralysis of limbs**						
No paralysis	2	4.3	45	95.7	Fishers exact test =	0.245
Single limb	0	0	18	100.0		
Hemiplegia/Quadriplegia	12	4.0	286	96.0	*p* =	1.000

*General Certificate of Examination - Ordinary Level

**General Certificate of Examination - Advanced Level

***Sri Lankan Rupees

Table 5 revealed that even though the men received a relatively higher percentage of an adequate level of essential post-stroke follow-up care services concerning the domains of treatment (*n* = 124, 61.4%), services to limit disabilities (*n* = 4, 2.0%,), services to modify lifestyle (*n* = 12, 5.9%), there is no significant inequity in women receiving an adequate level of care related to treatment services ( X^2^ = 0.48, df = 1,*p* = 0.51) and services to limit disabilities (X^2^ = 0.30, df = 1, *p* = 0.58) apart from services to modify lifestyle (X^2^ = 5.33, df = 1, *p* = 0.02) which recorded significantly high among men.

There was no inequity in coverage of essential post-stroke follow-up services by elderly patients compared to younger patients. Study participants who had completed secondary education received the highest percentage for adequate levels of services related to the monitoring of risk conditions (*n* = 5, 15.6%) although it is not significantly different to lower levels of education categories (X^2^ = 6.03, df = 3, *p* = 0.10). Concerning the treatment services, the majority of all 4 education level categories received adequate level essential post-stroke follow-up services and there is no significant inequity between age groups (X^2^ = 3.11, df = 3, *p* = 0.37). However, in relation to the services to modify lifestyle, none of the stroke survivors of the grade 0–5 group received an adequate level of services, while the GCE O/L passed group recorded the highest percentage with 7.4% (*n* = 5), and analysis estimates there is significant inequity observed ( Fisher’s Exact Test = 8.134,, *p* = 0.028) between education groups.

Concerning marital status, the not married group recorded a comparatively lower proportion of coverage of adequate services concerning all the stroke follow-up service domains. However, there is no significant inequity concerning any of the domains as revealed by the analysis.

In an analysis of income categories, the lowest income group reported the highest proportion of the adequate level of services for the domain of treatment (*n* = 70, 64.2%) and for the domain of ‘services to limit disabilities’ (*n* = 3, 2.8%). Even though the lowest income group received a lower proportion of adequate level of care related to domains; monitoring risk conditions (*n* = 11, 10.1%), services prevent complications (*n* = 6, 5.5%), services to modify lifestyle (*n* = 6, 5.5%), the difference between income categories are not revealed any significant inequities.

Coverage of adequate level of essential post-stroke follow-up care services concerning the domain of care on services to prevent complications, recorded significant inequity between study participants concerning the presence and type of paralysis of limb (Fishers Exact Test = 15.67, *p* = 0.00006). Apart from that no other domain of care revealed any significant inequity in coverage of essential post-stroke follow-up care services by presence and type of paralysis of limbs.

## Discussion

The World Health Organization defines Universal Health Coverage (UHC) as access to essential promotive, preventive, curative and rehabilitative health interventions for all, at an affordable cost. Achieving UHC is the cornerstone of Goal 3 on health and wellbeing of the Sustainable Development Goals to be reached by each country by 2030 [13]. Application of the UHC concept would mandate that post-stroke follow-up care services be made accessible to all stroke survivors in an equitable manner at an affordable cost without discrimination.

Defining essential post-stroke follow-up care services is complex owing to the wide variation in clinical presentation and underlying conditions of stroke. Our study developed a tool to assess the receipt of essential post-stroke follow-up care for stroke survivors at the end of the first six months following a stroke and evaluated whether it was equitably delivered to stroke survivors in the most urbanised province in Sri Lanka that includes its capital.

Although most stroke survivors (93.95%) had had their blood pressure measured and documented at least once during the first six months of follow-up, only one-fourth had been adequately monitored for blood pressure monthly. This, and the inadequate monitoring of blood glucose and lipids may be a result of the lack of uniformly agreed / National clinical guidelines/referral pathways in the post-stroke period and deficiencies in the existing laboratory services in the public health sector. coverage of secondary prophylaxis with antiplatelet medications (97.2%) and treatment for hypertension (73.9%) and diabetes (64.1%) was comparable to a previous hospital-based study conducted in Sri Lanka among 1000 patients who suffered from myocardial infarction and stroke which found that 76.9% were on aspirin, 19.6% were on statins and 44.2% and 39.2% were prescribed with beta-blockers and ACE Inhibitors respectively [14]. In comparison, data from developed regions have been variable. Provision of antiplatelet treatment in a study conducted in Ireland among 256 ischemic stroke survivors at 6 months post-stroke was higher [15] but in the United Kingdom, only 25.6% of the men and 20.8% of women were receiving secondary prevention drugs indicating that even in developed countries secondary prevention treatment services may be deficient [16].

This study further identified that services to limit disabilities and services to prevent complications were adequately received by less than 5% of the study population, indicating that the focus on tertiary prevention or rehabilitation of the stroke survivor is inadequate in Sri Lanka. In a study conducted in the USA, among 6206 stroke survivors, 75.7% had received rehabilitative therapy and 30.7% received restorative nursing care [17] while in a study conducted in Taiwan, only 34.1% of stroke survivors utilise long-term follow-up care related to limiting disabilities using institutional or community-based care services [18]. Qualitative studies conducted in developed countries such as Canada and developing countries such as Malaysia have identified that the lack of individualised care, lack of physiotherapy, occupational therapy, and speech and language therapy services for stroke survivors was due to resource constraints [19, 20]. These inadequate levels of coverage of services could be due to the lack of availability of rehabilitation services due to inadequate infrastructure as well as inadequate numbers of trained paramedical staff such as physiotherapists and occupational therapists within the public health system. In the Western province, public sector hospital beds available for rehabilitation care services were 256 out of a total of 23,580 hospital beds. The combined availability of physiotherapists, speech therapists and occupational therapists in the Western province was 0.64 per 10,000 persons. The annual rate of production of related human resources in the public sector is low. In 2019, the ministry of health trained 80 physiotherapists and 20 occupational therapists [21]. Only 0.1% of the total health budget was spent on rehabilitative care services [22]. These indicate a lack of prioritization of rehabilitative care services in Sri Lanka. Comparatively, the observed high coverage of treatment care services could be attributed to the availability of medical offices in the public sector as well as the private sector from the primary care level to the tertiary care level in this province. The well-established drug distribution mechanism of the ministry [23] and availability of free prescribed medications and relatively affordable medicines in the private sector [24] contribute to stroke survivors having access to the prescribed medicines. The non-availability of publicly available disable friendly transport mechanisms for stroke survivors could have affected their access to limitedly available rehabilitation care services [25, 26]. Even though some healthcare institutions have taken steps to increase the physical accessibility for stroke survivors by improving the built environment still health sector generally lacks overall accessibility [27]. Although some of these services can be provided to all stroke survivors through a trained, skilled/semi-skilled staff member in the stroke care team, no policy decisions have been taken for task-shifting or task-sharing among health care providers to overcome these critical service gaps. Despite policy directives to organise primary healthcare-based integrated care systems, the lack of implementation of these affects the service coverage of domains of care related to the management of stroke [23]. The requirement of an integrated health care model for stroke care services in Sri Lanka had been highlighted by previous researchers as well [28]. The necessity for rehabilitative care teams is also highlighted in the National Guidelines for Rehabilitative care services, which are yet to be implemented. These policy reforms still lack directives for domiciliary care services especially targeting economically vulnerable groups. However, options are available for the health policy makers to re-strategize the use of field-level preventive health staff to overcome some challengers in provision of stroke follow up care services. They could be trained to ensure integration of services between different levels and different providers and also re-trained to provide some selected follow up care services as they lacks up to date competencies related to stroke follow up care [29]. The major obstacle for this will be investments in improving available numbers in health cadres such as public health midwives and currently 97% of their time is allocated to Maternal and Child Health services [30].

The assessment of coverage of essential post-stroke social support services showed that stroke survivors who received information and benefits from other institutions were very low (3.6%), which could be due to the majority of the health care providers being unaware of the availability of such services for stroke survivors. A drop in employment of 13.2% after 6 months of stroke, could be due to the decisions of stroke survivors to ‘stop’ working or employers being reluctant to continue them in the job. The lack of exchange of information between the health sector and the social services may have hindered the tilization of available resources from other government agencies for stroke survivors.

To the best of our knowledge this is the first attempt to explore the inequity of stroke follow-up care services in Sri Lanka. The disaggregation of coverage of services by equity dimensions identified that those less than 60 years received a higher proportion of ‘adequate level’ of stroke follow-up care services related to the monitoring of risk factors, indicating that older patients were ‘left behind’. The difference observed for coverage of services to prevent complications by the presence and type of limb paralysis might be due to a lack of availability of suitable transport facilities for stroke survivors. Men receiving a significantly higher level of services to modify lifestyle could be related to the presence of higher levels of modifiable risk factors among men. Additionally, the O/L pass -educational level group receiving significantly higher level of care in the domain of services to modify lifestyle could be due to relatively higher level of health literacy among this group. No other inequities were observed concerning the coverage of essential post-stroke follow- up care services by sex, education level, marital status, family monthly income level and presence and type of paralysis of limbs. The declared family income revealed that 83.3% of the study participants belong to the LKR 50,000 category. This resulted in a more homogenous study sample in terms of family income, and this could have led to no significant equity variations between groups. The low availability of essential post-stroke care services both in the public and private sector could also explain the minimal inequities observed. Previously a group of researchers assessed the inequalities of diabetes mellitus and its risk factors in a suburban district of Sri Lanka. They also failed to observe any relationship between socioeconomic status and the prevalence of diabetes [31].

Our study has some limitations. Firstly, we enrolled study participants only from the public sector health care institutions. Even though the private health sector provides 5%-10% of in- patient care, having study participants from both type of institutions could have identified more variation especially regarding the equity of access to services. Secondly, the proportion of stroke survivors who received the essential post-stroke follow-up care services was determined based on documented evidence and by recall. Hence, some services received would neither have been documented nor recalled. Additionally, we measured the coverage of essential post-stroke are services at least once during six-month period for 49 services that were categorised under six domains of care. Therefore, study participants may have received these services at least once, but the quality and frequency of services could have been different between high-income and low-income categories which we did not measure in this study.

## Conclusion

In conclusion, other than treatment services, a majority of the stroke survivors did not receive an adequate level of essential post-stroke follow-up care services, related to monitoring of risk conditions, services to limit disabilities, services to prevent complications, lifestyle modifications and supportive services as assessed at the end of six-months following the stroke incident. There were no apparent inequities in receiving post-stroke follow-up care services in the Western Province of Sri Lanka. As a follow-up study of a cohort of stroke survivors, this study was able to describe the inadequacies of services received by the stroke survivors in their follow-up period. Health policy makers should review the existing health service delivery mechanisms and assess the required human resources for health, to ensure people are receiving the required promotive, preventive, treatment, and rehabilitative health services with sufficient quality without causing financial hardships to achieve Universal Health Coverage.

## Data Availability

The datasets used and/or analysed during the current study available from the corresponding author on reasonable request.
